# A Comparative Study of the Biodurability and Persistence of Gold, Silver and Titanium Dioxide Nanoparticles Using the Continuous Flow through System

**DOI:** 10.3390/nano13101653

**Published:** 2023-05-16

**Authors:** Odwa Mbanga, Ewa Cukrowska, Mary Gulumian

**Affiliations:** 1 Molecular Sciences Institute, School of Chemistry, University of Witwatersrand, Private Bag X3, Johannesburg 2050, South Africa; 367076@students.wits.ac.za (O.M.); ewa.cukrowska@wits.ac.za (E.C.); 2 Water Research Group, Unit for Environmental Sciences and Management, Northwest University, Private Bag X6001, Potchefstroom 2520, South Africa

**Keywords:** gold and silver nanoparticles, titanium dioxide nanoparticles, biodurability, persistence, dissolution kinetics, simulated fluids

## Abstract

The potential for nanoparticles to cause harm to human health and the environment is correlated with their biodurability in the human body and persistence in the environment. Dissolution testing serves to predict biodurability and nanoparticle environmental persistence. In this study, dissolution testing using the continuous flow through system was used to investigate the biodurability and persistence of gold nanoparticles (AuNPs), silver nanoparticles (AgNPs) and titanium dioxide nanoparticles (TiO_2_ NPs) in five different simulated biological fluids and two synthetic environmental media to predict their behaviour in real life situations. This study examined the physicochemical properties and agglomeration state of gold, silver and titanium dioxide nanoparticles before and after dissolution tests using three different techniques (UV-vis, XRD and TEM). The UV-vis spectra revealed that all three nanoparticles shifted to higher wavelengths after being exposed to simulated fluids. The titanium powder was found to be mixed with both rutile and anatase, according to XRD examination. The average diameter of gold nanoparticles was 14 nm, silver nanoparticles were 10 nm and titanium dioxide nanoparticles were 25 nm, according to TEM images. The gold and silver nanoparticles were observed to be spherical, but the titanium dioxide nanoparticles were irregular in shape, with some being spherical. The level of dissolved nanoparticles in simulated acidic media was higher in magnitude compared to that dissolved in simulated alkaline media. The results obtained via the continuous flow through dissolution system also displayed very significant dissolution rates. For TiO_2_ NPs the calculated half-times were in the range of 13–14 days, followed by AuNPs ranging between 4–12 days, significantly longer if compared to the half-times of AgNPs ranging between 2–7 days. AuNPs and TiO_2_ NPs were characterized by low dissolution rates therefore are expected to be (bio)durable in physiological surroundings and persistent in the environment thus, they might impose long-term effects on humans and the environment. In contrast, AgNPs have high dissolution rates and not (bio)durable and hence may cause short-term effects. The results suggest a hierarchy of biodurability and persistence of TiO_2_ NPs > AuNPs > AgNPs. It is recommended that nanoparticle product developers should follow the test guidelines stipulated by the OECD to ensure product safety for use before it is taken to the market.

## 1. Introduction

The manufacturing, production and application of nanoparticles is ever increasing and making a profound impact [1,2]. For example, gold nanoparticles (AuNPs) are used in the medical field as drug delivery agents since they are biocompatible, easy to manipulate in size and shape and are chemically stable [3,4,5,6]. Whereas silver nanoparticles (AgNPs) owing to their antimicrobial properties are used in the food and cosmetics industries [7,8]. Titanium dioxide nanoparticles (TiO_2_ NPs) are extensively used as food colourants, nutritional supplements and for food packaging materials [9]. This is due to their ability to filter UV radiation, have antimicrobial properties and are excellent inhibitors of corrosion [10]. Their extensive use in consumer products has resulted in humans being increasingly exposed and they are also released to the environment in many ways including waste disposal [11]. However, much is still unknown about the effects of nanoparticles on human health and the environment. Many discussions are currently ongoing as to whether exposure of NPs to the ecosystem (i.e., plants and animals, humans and the environment) may be conceived as harmful or not [1].

The application of nanoparticles offers a wide range of benefits; however, unlocking this potential requires a responsible and co-ordinated approach to ensure that potential challenges are being addressed in parallel with the development and use of nanotechnology [12]. The traditional testing and assessment methods used to determine the safety of traditional chemicals are not necessarily applicable to NPs [13,14]. The concept of safe by design has been used in a variety of industries to identify potential risks and minimize those risks early in the technological development process. Biotechnology, crop breeding and drug design are examples of industries [15]. To ensure that safety and sustainable usage of nanoparticles is a key priority, safe by design concepts and methodologies used in these industries should also be used in nanotechnology and the development of advanced and smart materials [15]. In this study dissolution was used to assess the biodurability and persistence of AuNPs, AgNPs and TiO_2_ NPs to gain a better understanding of their effects on human health and the environment. This is because many studies are concerned mostly with the assessment of toxicity, a challenging but yet unaddressed issue of nanoparticles is their biodurability, which is the tendency to resist dissolution and biodegradation within biological and environmental surroundings [16]. Whereas persistence is the capacity of a substance, particle or fibre to remain unchanged in the environment for a very long time [17,18]. Dissolution tests provide a measure of nanoparticles biodurability and persistence, which can provide useful information about their acute and long-term toxicity as well as the particles’ pathogenicity [16]. For example, if a particle dissolves rapidly, it is more likely to cause short-term health effects and its impact on the environment can manifest faster [19]. However, particles that dissolves slow are biodurable and hence may cause both short-term and long-term health effects and show high environmental persistency [12]. For metal-containing nanomaterials, the release of metal ions is thought to be the primary cause of any induced toxicity [12,16,18]. Therefore, it is of utmost importance to study dissolution to better understand the behaviour of nanoparticles in real life situations.

A proper understanding of the safety of nanoparticles requires information on their biodurability in physiological surroundings and persistence in the environment. Currently, several research studies have been conducted on the risk assessment and safety of nanoparticles. For example, a study conducted by Avellan et al. [20] predicted the fate of AuNPs in mesocosms freshwater wetland to simulate aquatic environments and found that some plants can oxidize AuNPs thereby releasing Au^+^ ions. Other data in the literature have reported on the biodistribution and accumulation of AuNPs in several cell lines and models and the factors identified to influence their toxicity are surface charge and functionalization, size and shape of AuNPs [3,21,22,23]. Furthermore, long-term and short-term dissolution studies of AgNPs have been conducted by numerous researchers [1,24,25,26,27]. Factors which influence dissolution include agglomeration state of nanoparticles, ionic strength of the media and particle surface functionalization [24,28,29,30,31]. Even though TiO_2_ NPs are considered insoluble therefor undergo negligible dissolution in biological and environmental media, there is sizable research that has been conducted which elucidates their dissolution in media [32,33,34,35].

However, a lot of these studies do not thoroughly elucidate the dissolution kinetics of particles. Little is known about how long it would take for nanoparticles to disintegrate in the body and the environment, and how fast that process occurs. This current research study is concerned with addressing these issues. Therefore, there is a need to elucidate the biodurability and persistence of nanoparticles to gain a better understanding of their safety and predict their behaviour in real life situations. In this work we predicted the biodurability and persistence of AuNPs, AgNPs and TiO_2_ NPs in a wide range of in five different simulated biological fluids and two synthetic environmental media to predict their behaviour in real life situations. These parameters were predicted by studying the dissolution kinetics, including the dissolution rates, rate constants, order of reaction and half-times of AuNPs, AgNPs and TiO_2_ NPs to predict their behaviour in physiological and environmental conditions.

It is hypothesized that since nanoparticles are utilised in a wide variety of consumer products, there is concern regarding potential exposure. If these nanoparticles are released into the environment, they may cause negative effects on both the environment and biological organisms. It is expected that when subjected to simulated acidic fluids, the nanoparticles will release ions, whereas in neutral simulated fluid, the nanoparticles will be stable. Short-term toxicity could be due to either the particles or the ions released by them. Longer half-time nanoparticles, on the other hand, will have more severe long-term consequences.

## 2. Materials and Methods

### 2.1. Characterization of Gold, Silver and Titanium Dioxide Nanoparticles

The three different types of nanoparticles namely AuNPs, AgNPs and TiO_2_ NPs were tested for their biodurability and persistence through investigating their dissolution behaviour and dissolution kinetics. The dissolution tests were conducted using the continuous flow-through system in simulated biological fluids and synthetic environmental media to mimic body fluids and environmental media. The 14 nm in diameter AuNPs were obtained in three different types and provided by MINTEK (Randburg, South Africa). The first type was the citrate stabilized gold nanoparticle (AuNPs-cit) with the concentration of about 3.8 nM, followed by PEGylated carboxyl functionalized gold nanoparticle (AuNPs-COOH) whose concentration was 4.0 nM and the last one was the PEGylated amine functionalized gold nanoparticles (AuNPs-NH_2_) whose stock solution had a concentration of 3.0 nM. AgNPs were purchased from (Sigma Aldrich Johannesburg, South Africa) in the size of 10 nm in diameter with the concentration of 0.02 mg mL^×1^ suspended in a 1% sodium citrate solution as a stabilizer. For TiO_2_ NPs, a unit of standard reference material (SRM) 1898 was purchased from the National Institute of Standards and Technology (NIST, Gaithersburg, MD, USA). All the nanoparticle suspensions were prepared under sterile conditions. Transmission electron microscope (TEM) (JOEL Ltd. JEM-2100) (Lireweg, The Netherlands) analyses were performed before and after dissolution studies to monitor the morphological changes in the nanoparticles upon exposure to simulated fluids. The Specord 50 Analytik Jena Ultraviolet-Visible spectrophotometer (UV-is) (Analytik Jena GmbH+Co. KG, Jena/Germany) was used to determine the agglomeration and aggregation state of NPs in simulated media at various wavelengths before and after dissolution experiments. Titanium dioxide nanoparticles were further characterized with an X-ray diffractometer the PANayltical X’Pert Pro powder diffractometer instrument (Malvern, United Kingdom) was used to determine their crystalline structure and to confirm whether they existed in the anatase or rutile crystal phase. This instrument was fitted with 1D X’Celerator detector, 10 mm programmable divergence slit and sample spinner (Spinner PW3064) with a rotation time of 1 s. The X-ray radiation source was Cu Kα (λ = 0.15405 nm) tube, operating at 40 kV and 40 mA conditions. The measurement was carried out under Gonio scan axis with continuous scan type, step size, scan step time and 2θ range of 0.0170°, 2θ, 87 s and (5 to 90°), respectively. The P-XRD sample was transferred onto the low background silicon sample holder. After the X-ray measurements, raw data were interpreted by using High Score (Plus) software with ICDD PDF-4^+^ 2019 database. The concentrations of dissolved Au, Ag and Ti ions were obtained using inductively coupled mass spectrometer (ICP-MS) (Agilent Technologies, 7700 series ICP-MS, Santa Clara, CA, USA).

### 2.2. Preparation of Simulated Fluids

Nanoparticles can enter the human body via various routes, the focus of this present research study was therefore exposure via inhalation, ingestion, intravenous and environmental exposure through waste disposal. Subsequently, simulated phagolysosomal fluid (PSF) and Gamble’s fluid (GF) were chosen to represent lung fluids found in cellular lysosomes and deep within the lungs at pH 4.5 and pH 7.4, respectively. Whereas gastric fluid (GIF) and intestinal fluid (IF) were representative of stomach fluids at pH 2.0 and pH 7.5, respectively. Lastly, blood plasma (BP) at pH 7.2 which is a fluid that carries blood components throughout the body. The synthetic environmental media of choice were freshwater (FW) and seawater (SW). The preparation of all the simulated fluids was adopted from the procedure presented by Innes et al. [16] and Marques et al. [36] using the reagents listed in Table 1. Synthetic environmental media were prepared following the procedure recommended by the United States (U.S) Environmental Protection Agency (EPA). These reagents were dissolved in 5 L of ultrapure milli-Q water with a resistivity of 18.2 MΩ·cm in the order given in Table 1, and the pH was adjusted with either 1 M hydrochloric acid or 1 M sodium hydroxide. A 25 µL alkylbenzyldimethylammonium chloride (ABDC) the anti-fungal agent was added to each 5 L container to preserve the simulated biological fluids and synthetic environmental media.

### 2.3. Continuous Flow-Through Dissolution Procedure

**Figure 1:**
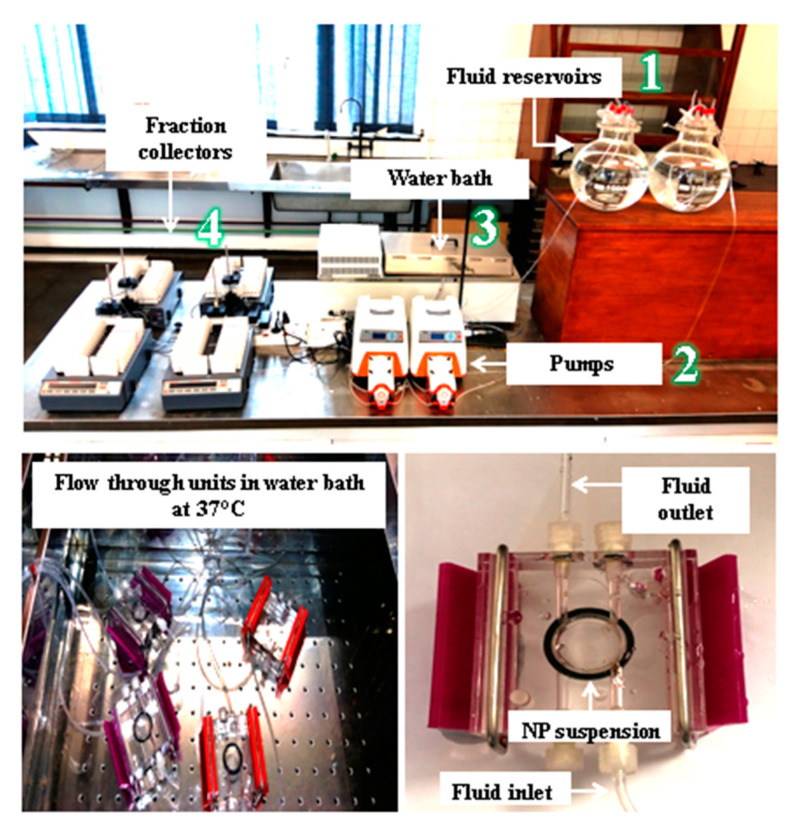
Continuous flow-through dissolution system protocol.

The continuous flow-through dynamic method of dissolution testing protocol shown in Figure 1 used in this study was adapted from Keller et al. [37] and Koltermann-Jülly et al. [38] with minor changes to match the specifications of nanoparticles. This dissolution protocol was specifically selected because it is regarded to be more reflective of dissolution occurring in biological and environmental surroundings. It is therefore recommended to avoid achieving an equilibrium that would restrict dissolution. A volume of 2 mL of gold and silver nanoparticles were drawn from the nanoparticle stock solutions and transferred into small centrifuge tubes. These were centrifuged at 13,000 times gravity for 30 min to pre-concentrate the samples which formed pellets. The pellets were transferred separately into the lower chamber of the flow through units. The flow through units containing the pellets were then filled with simulated fluids to create a nanoparticle suspension. TiO_2_ NPs were in a powder form therefore, a mass of 1 mg of titanium dioxide nanoparticle powder was weighed onto a membrane and was also transferred into the lower chamber of the flow through unit which was also filled with simulated fluid forming a nanoparticle suspension. An o-ring membrane holder was placed on top of the flow through unit containing the NP suspensions which was then sealed with the 3.5 kD membrane. The three separate flow through units containing AuNP, AgNP and TiO_2_ NP suspensions were then closed with a membrane (Spectrum/Por 3—Standard RC Discs—MWCO: 3.5 kD–33 mm^2^) pore size to only permit the movement of dissolved ions. A small pore size membrane was carefully selected to ensure that all the nanoparticles were kept inside the flow through units whilst only permitting the dissolved ions to diffuse into the bulk fluid. A second flow through unit (upper chamber) was placed on top of the membrane sealed lower chamber and tightly closed to only permit the movement of dissolved ions into the fraction collectors. The three separate flow through units were simultaneously submerged in a water bath maintained at 37 °C to mimic physiological conditions and room temperature 25 °C for synthetic environmental fluids. The simulated fluids from the fluid reservoir were pumped through the flow through units using the peristaltic pump at 8 mL/h and the eluate containing dissolved ions were continuously collected by the fraction collectors. The concentration of released ions from the eluate were analysed by ICP-MS to determine the level of dissolved ions of gold, silver and titanium. The programmable sampler drew 8 mL/h of the eluate. The dissolution experiments were conducted over a period of 10 days and triplicate samples were taken and measured. Samples were collected in 30 min interval for the first 4 h and once a day for the next 10 days. Sampling times were 0 h, 0.5 h, 1 h, 1.5 h, 2 h, 2.5 h, 3 h, 3.5 h and 4 h. From day 2 to day 10 samples were collected at 24 h, 48 h, 72 h, 96 h, 120 h, 144 h, 168 h, 192 h and 2016 h. Reported in the results section is an average of the three measurements.

### 2.4. Determination of the Kinetic Parameters

The dissolution of nanoparticles follows the first order reaction kinetics and involves the mass transfer rate process whereby the solute is transported from the nanoparticle surface to the bulk fluid surrounding the nanoparticles. The rate of solute liberation and transport from the nanoparticle surface is calculated using the dissolution kinetics model described below. To determine the dissolution rate constant and half-time of nanoparticles in the current research, the dissolution data were fit to a first order kinetic model previously described by Keller et al. [39] and Koltermann-Jülly et al. [38] using the following equation:

(1) $$M_{dissolved}\left(T\right)=\frac{m(ENM)}{m(metal\;ion)}\times \sum_{i=0}^{T}c_{i}\left(ion\right)\times V_{i}\times {∆t}_{i}$$

where $M_{dissolved}\left(T\right)$ is the mass of the dissolved nanoparticles, $m\left(ENM\right)$ is the initial mass of the nanoparticles weighed before the commencement of the dissolution experiments, m(metal ion) is the mass of dissolved nanoparticles obtained at different sampling time points, $c_{i}\left(ion\right)$ is the concentration of dissolved ions obtained at a specific sampling time point, $V_{i}$ is the volume sampled at different times and ${∆t}_{i}$ is the different time interval where samples were collected to determine the amount of dissolved ions. Equation (1) gives us the rate of mass removal and the dissolution rate k is obtained using Equation (2) which is calculated as follows:

(2) $$M_{solids}\left(T\right)=M_{0}-M_{dissolved}(T)$$

From Equation (2) the mass of nanoparticles remaining can be determined where $M_{solids}\left(T\right)$ is the mass remaining after dissolution has occurred, $M_{0}$ represents the initial mass of the before dissolution and $M_{dissolved}\left(T\right)$ is the mass dissolved at different sampling time points. Equation (2) allows us to determine the dissolution rate k which is calculated using the following equation:

(3) $$k=ln\{M_{0}/M_{solids}(T)\}/(\mathrm{SSA}(T)\times T)$$

where k is the dissolution rate, SSA represents the initial surface area of the nanoparticles before dissolution and T is the time taken for the duration of the dissolution experiments. The initial surface area provided in Table 2 was used to calculate the half-time of nanoparticles to predict their duration in biological and environmental surroundings using the following equation:

(4) $$t_{1/2}=\frac{\ln2}{k*SSA}$$

To calculate the mass of dissolved ions and account for the molar masses of the nanoparticles and detectable metal ions, we multiplied the measured ion concentration of each eluate by the eluted volume. This allowed us to calculate the percentage mass of remaining nanoparticles during the sampling time intervals.

### 2.5. Statistical Analysis

The data on dissolution are presented as the mean standard deviation of at least three independent measurements. To determine significant differences in the dissolution kinetics of AuNPs, AgNPs and TiO_2_ NPs in various simulated body fluids and synthetic environmental media, a multiple variable ANOVA analysis was performed using RStudio version 1.2 software. *p* < 0.05 was considered statistically significant.

## 3. Results

### 3.1. Physichichemical Properties of AuNPs, AgNPs and TiO_2_NPs

Investigating the biodurability and persistence of nanoparticles requires a thorough and accurate characterization of the particles’ physicochemical properties which can in turn be linked to their dissolution behaviour. In the present study, UV-vis, XRD and TEM were used to characterize, assess and monitor morphological changes and agglomeration states of AuNPs, AgNPs and TiO_2_ NPs before and after the dissolution experiments. Table 2 shows the physicochemical characterization of AuNPs, AgNPs and TiO_2_ NPs.

Generally, the UV-vis spectra of gold, silver and titanium dioxide nanoparticles have a localized surface plasmon resonance peaks at 520 nm, 400 nm and 300 nm, respectively [40,41,42,43]. This was confirmed by the UV-vis characterization of these nanoparticles before the dissolution experiments as shown in Table 2. After exposure to simulated fluids there was an observable shift to higher wavelengths for all the three nanoparticles. Interestingly this red shift for AuNPs was functional group specific. For example, -AuNPs-cit shifted to 547 nm followed by AuNPs-COOH at 540 nm then lastly AuNPs-NH_2_ shifted to 540 nm. These subtle differences are likely due to that the functionalized AuNPs are coated with polyethylene glycol (PEG) then functionalized with the carboxyl and amine functional groups. Consequently, PEG provides electro steric stabilizing thereby preventing the particles from combining to form agglomerates as a result, they remain monodispersed [44,45]. However, the citrate on the citrate stabilized AuNPs is just a stabilizing agent which can be easily displaced from the NP surface as a result it is easier to from agglomerates once the stabilizing agent is removed. For AgNPs there was an observable shift to higher wavelength (450 nm) for particles in contact with neutral media such as blood plasma, intestinal fluid, Gamble’s fluid and freshwater. This indicates that after a prolonged exposure of silver nanoparticles to these simulated fluids the particles physically coalesce to form larger particles. Generally larger particles absorb light at higher wavelength than smaller particles hence there was an observable shift to higher wavelengths for the agglomerates. TiO_2_ NPs exhibited a similar trend whereby there was a shift to higher wavelengths (320 nm) after exposure to simulated fluids indicative of particle aggregation as the time of exposure to simulated fluids increased. For AuNPs and AgNPs this red shift in wavelength was due to particle agglomeration whereas, for TiO_2_ NPs the major cause of the shift was formation of particle aggregates. Particle aggregation leads to a reduced surface area because particles combine to form a union of larger particles through weak Van der Waal forces [35,46,47]. As a result, the nanoparticle absorbs UV-light at a much higher wavelength as shown in Table 2 after dissolution experiments.

### 3.2. XRD Characterization of TiO_2_ NP Powder

**Figure 2:**
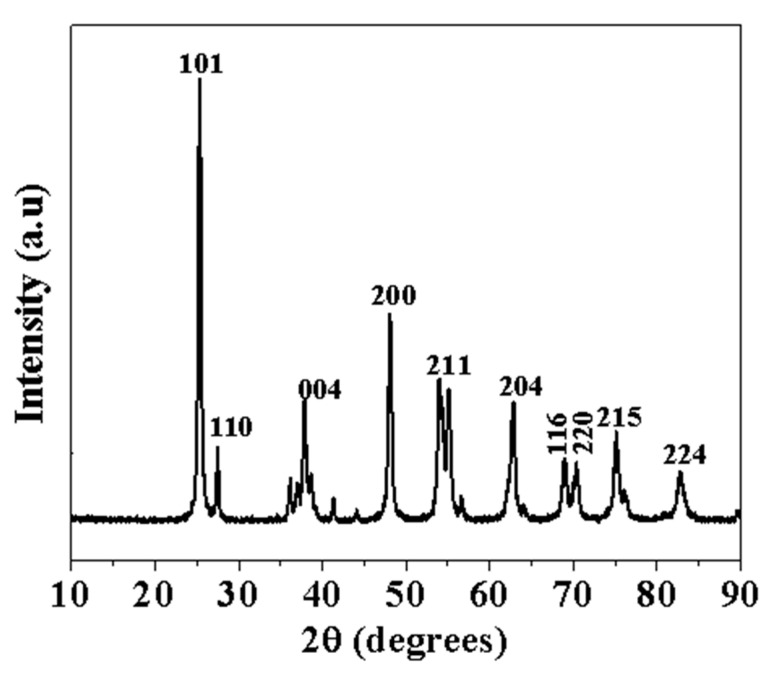
X-ray diffraction pattern of TiO_2_ NPs powder.

The TiO_2_ nanoparticle powder was examined using XRD to assess its crystallographic phase, whether it was rutile, anatase, or a combination of both. The XRD pattern of the nanoparticles can be seen in Figure 2, with the peak positions at 2θ and their Miller indices. The TiO2 XRD data demonstrated very sharp peaks. The strong diffraction peaks exhibited by the XRD pattern at angles 25°, 37°, 47°, 55°, 62°, 68°, 70°, 75° and 82° correspond to Miller indices of (101), (004), (200), (211), (204), (116), (220), (215) and (224) plane, respectively. The major component of the TiO2 NPs sample was confirmed to be anatase. However, there was a minor presence of rutile which is represented by the peaks corresponding to (110) and (211) planes.

### 3.3. TEM Characterization of AuNPs, AgNPs and TiO_2_ NPs

**Figure 3:**
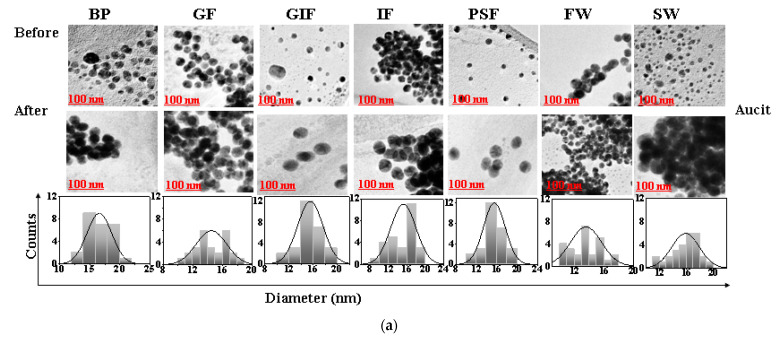

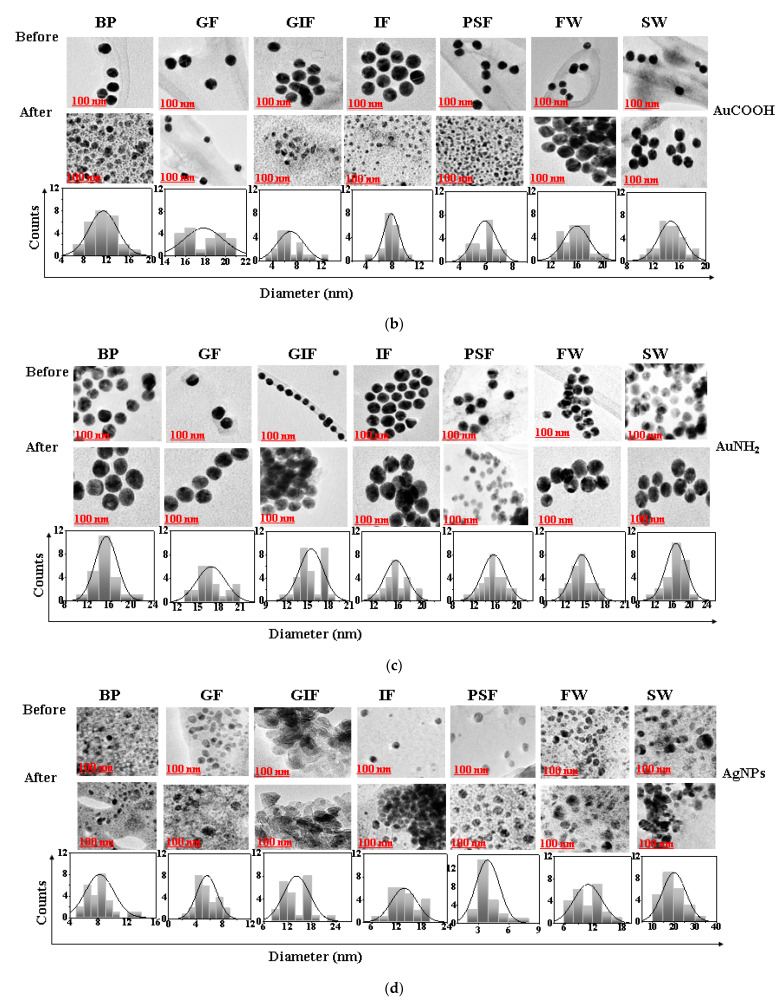

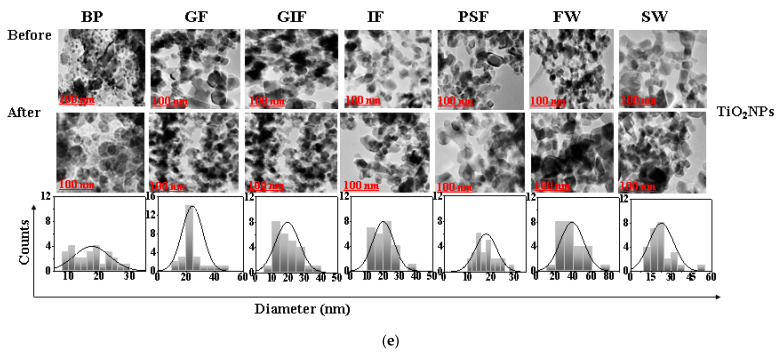
TEM images of cit-stabilized AuNPs (**a**); COOH-AuNPs (**b**) NH_2_-AuNPs (**c**), AgNPs (**d**) and TiO_2_ NPs (**e**) in simulated biological and environmental fluids before and after dissolution tests. BP, GF, GIF, IF and PSF are simulated biological fluids for blood plasma, Gamble’s fluid, gastric fluid, intestinal fluid and phagolysosomal fluid, respectively. FW and SW are synthetic environmental fluids for freshwater and seawater, respectively.

TEM was used to investigate the morphological changes in AuNPs, AgNPs and TiO_2_ NPs in simulated fluids before and after the dissolution experiments. The TEM images of AuNPs-cit, AuNPs-COOH, AuNPs-NH_2_, AgNPs and TiO_2_ NPs in simulated fluids are shown in Figure 3a–e respectively.

The average nanoparticle diameter measured using Image J software (National Institute of Health, version no Java1.8.0_172) were 14 ± 2.8 nm for citrate stabilized AuNPs, 14 ± 2.3 nm for COOH-AuNPs and 14 ± 1.7 nm for NH_2_-AuNPs. Whereas the AgNPs had a size diameter of about 10 ± 0.8 nm which was smaller than that of TiO_2_ NPs with the size of 25 ± 3.1 nm. The gold and silver nanoparticles were spherical in shape as shown in Figure 3a–d. However, TiO_2_ NPs were irregular in morphology, and some were spherical as seen in Figure 3e. Among the AuNPs the citrate stabilized AuNPs tended to form multiple single particle clusters after exposure to simulated fluids. In contract, the pegylated carboxyl and amine functionalized gold nanoparticles (AuCOOH) and (AuNH_2_) remained monodispersed throughout the duration of the dissolution experiments. The Peg polymer coating present on the surface of functionalized gold nanoparticles provides steric stability which prevents the particles from colliding together to form clusters. Additionally, polymers are always present in the suspension system for steric stabilization, and they adsorb onto the particle surface, resulting in an additional steric repulsive force. Silver nanoparticles were spherical in shape and monodispersed. The morphological analysis of the TiO_2_ nanopowder by TEM (Figure 3e) showed high degrees of particle aggregation in all simulated fluids despite the differences in chemical composition, pH and ionic strength of the simulated fluids [48,49]. The formation of nanoparticle aggregates is due to Van der Waals interactions on the nanoparticle surface. During particle–particle interactions, if the force of attraction far exceeds the repulsive forces, then particles will tend to stick together to form aggregates [29,50]. The formation of nanoparticle aggregates can hinder the dissolution process by reducing the exposed surface area of the particle [51]. Additionally, particle aggregation can introduce a kinetic hindrance effect to the diffusion process thereby significantly reducing chances of dissolution [52].

### 3.4. Dissolution Curves of AuNPs, AgNPs and TiO_2_ NPs

**Figure 4:**
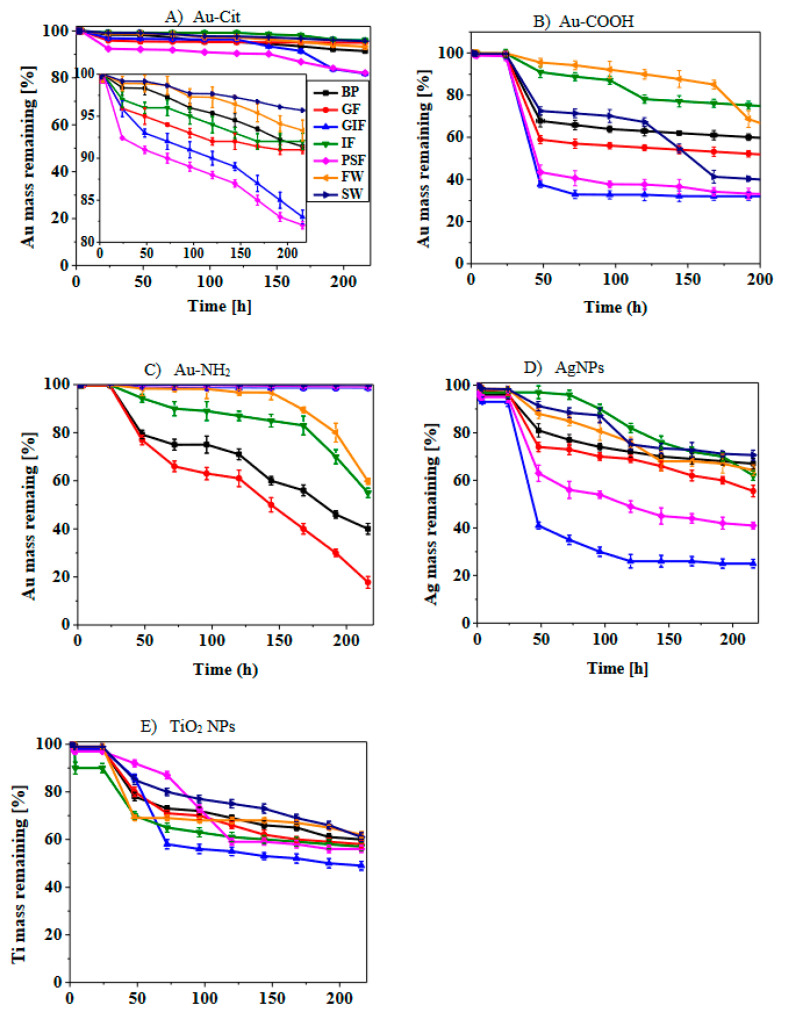
Dissolution profiles for cit-AuNPs (**A**); COOH-AuNPs (**B**); NH_2_-AuNPs (**C**); AgNPs (**D**) and TiO_2_ NPs (**E**) in simulated fluids. Simulated biological fluids are BP—Blood plasma, GF—Gamble’s fluid, GIF—Gastric fluid, IF—Intestinal fluid and PSF—Phagolysosomal fluid. Synthetic environmental media are FW—Freshwater and SW—Seawater.

Figure 4 presents the dissolution curves of AuNPs, AgNPs and TiO_2_ NPs in various simulated biological fluids and synthetic environmental media. The dissolution curves are reported as a percentage mass of nanoparticles that remained undissolved in the reaction vessel over a period of 10 days expressed as time in hours. This method was adopted from these researchers Koltermann-Jülly et al. [38] and Keller et al. [39].

There was no complete dissolution of all the nanoparticles in all the simulated fluids. Additionally, dissolution was gradual in all cases meaning the release of ions from all the nanoparticle surfaces commenced after 24 h. Of the three types of AuNPs, COOH-AuNPs showed the highest amount of dissolved Au^+^ ions with the maximum found in acidic media such as gastric fluid and phagolysosomal fluid. For example, from the starting mass of 1 mg, only 32% and 33% mass remained undissolved in simulated gastric fluid and phagolysosomal fluid, respectively. Over the period of 10 days, cit-AuNPs exhibited the lowest dissolution whereby the maximum dissolution occurred in simulated phagolysosomal fluid. In addition, 81% mass of the particles remained undissolved. However, for NH_2_-AuNPs, the dissolution was higher in alkaline media such as simulated blood plasma and Gamble’s fluid. The reason for high dissolution of these nanoparticles could be due to the presence of this compound in the simulated fluid which acts as a solubilizing agent and encourages formation of more soluble complexes [53]. This occurs via the complexation of the nanoparticles with these compounds thereby facilitating the liberation of these nanoparticle ions which ultimately diffuse into the bulk fluid [53]. Interestingly, cit-AuNPs showed limited dissolution compared to functionalized COOH-AuNPs and NH_2_-AuNPs. This demonstrates the effect of surface functionalization on dissolution.

AgNPs showed significantly higher dissolution compared to both AuNPs and TiO_2_ NPs. TEM images of AgNPs revealed a decrease in size diameter after the end of the dissolution experiments and this is evidence of dissolution. The nanoparticles started releasing ions after 24 h of exposure to simulated fluids. AgNPs dissolved in acidic simulated gastric fluid and phagolysosomal fluid within 48 h but took longer to dissolve in alkaline media such as Gamble’s fluid, intestinal fluid and blood plasma. Generally, when particles were exposed to simulated fluids, the dissolution was significantly lower in alkaline media than in acidic media. There was no observable plateau reached during dissolution because under continuous flow through conditions the equilibrium is not reached therefore, the particles keep releasing ions till the end of the dissolution experiments. These results are in agreement with those obtained by Keller et al. [39] where barium sulphate dissolved in phagolysosomal fluid after two years.

For TiO_2_ NPs the amount of dissolved Ti ions did not even reach 50% of the initial mass in all simulated fluids regardless of the differences in chemical composition and pH of simulated fluids. The dissolution profile of TiO_2_ NPs showed low dissolution in both alkaline and acidic media. From the physicochemical properties data provided and the TEM images in Figure 3, it can be observed that the surface area of TiO_2_ NPs is larger compared to that of AuNPs and AgNPs. Research has shown that particles with a larger surface area are less reactive than those with smaller surface area [54,55].

When comparing the dissolution profiles of the synthetic environmental fluids, synthetic seawater had lower dissolution of all three nanoparticles compared to freshwater. However, COOH-AuNPs submerged in seawater dissolved faster than those exposed to freshwater. Even though COOH-AuNPs dissolved faster in seawater, the dissolution of particles in seawater and freshwater was not statistically significant except AgNPs. Particles showed degrees of agglomeration in both waters, but in seawater this was enhanced by the high ionic strength of the media. This could be attributed to the high ionic strength of seawater and the presence of divalent cations in high concentrations [30,56]. The influence of ionic strength is further explained in the discussion section.

### 3.5. Dissolution Kinetics of AuNPs, AgNPs and TiO_2_ NPs

Dissolution kinetics are a crucial factor in determining the safety of nanoparticles, which affect the biodurability and persistence of particles in biological and environmental surroundings. The kinetic model presented in the materials and methodology section was used to determine the dissolution kinetics. Additionally, the biodurability and persistence of AuNPs, AgNPs and TiO_2_ NPs were estimated using the dissolution rates and half-times. The data are presented in Table 3. The dissolution rates were determined over a period of 10 days in different simulated biological fluids and synthetic environmental media. From Table 3 it was observed that the dissolution rates of AgNPs were significantly higher (*p* < 0.05) and half-times were shorter in all media compared to those of AuNPs and TiO_2_ NPs. For example, the half-times of AgNPs range between the period of 2–7 days, whereas the half-times of AuNPs and TiO_2_ NPs fall within the range of 4–12.5 days and 13.5–14.4 days, respectively. TiO_2_ NPs had longer half-times regardless of the pH and chemical composition of simulated fluids. Generally, dissolution rates of particles in simulated gastric fluid and phagolysosomal fluid were higher than those of alkaline media such as blood plasma and intestinal fluid with the exception of NH_2_-AuNPs. The dissolution rate constants in Table 3 show that the dissolution rates of AuNPs increase in the rate of COOH > NH_2_ > citrate. Accordingly, the aggregation follows the inverse order and is influence by the protection of the gold core by the polyethylene-glycol in the case of COOH and NH_2_ functionalized nanoparticles. The high dissolution rate of amine functionalized nanoparticles in simulated blood plasma, Gamble’s fluid and intestinal fluid could be due to the interaction of the amine group and components of simulated fluids leading to the formation of more soluble complexes. In the case of synthetic environmental fluids, dissolution in simulated freshwater was faster than in seawater for all the nanoparticles. This is due to the ionic strength of seawater. Furthermore, the dissolution rates data indicate that the rate and extent of dissolution depends on the pH of simulated fluids, chemical composition of the simulated fluids, nanoparticle surface area and aggregation state and are nanoparticle specific. The observed dissolution rates of the nanoparticles in this study followed the order AgNPs > AuNPs > TiO_2_ NPs. These results were corroborated by those obtained by Koltermann-Jülly et al. [38]; Keller et al. [39]; Braun et al. [54]; Shinohara et al. [57].

## 4. Discussion

The high dissolution rates of silver nanoparticles can be attributed to the nanoparticle surface exposed in the simulated fluids. From the TEM images it is evident that these silver nanoparticles are monodispersed and not agglomerated meaning there is a larger particle surface area exposed and this results in enhanced interaction between the components of the simulated fluids and silver nanoparticles thereby encouraging dissolution. However, this is not the case for titanium dioxide nanoparticles, as most of the particles are highly aggregated thereby minimizing the exposed surface area. The effect of poor dissolution because of particle aggregation is dramatically enhanced for poorly soluble particles such as TiO_2_ NPs [53,57]. This is because smaller particles have many reactive atoms on the nanoparticle surface and ready to interact with the components of the simulated fluids. In addition, for this reason, TiO_2_ NPs take longer to release Ti ions, therefore have low dissolution. Additionally, the TEM images in Figure 2e show high degrees of TiO_2_ particle aggregation. In addition, particle aggregation has been shown to slow dissolution [17,58]. This is because as the particles combine to form clusters, this significantly reduces the particle surface area available for dissolution. As a result, the diffusion of Ti ions from the surface is inhibited thereby limiting dissolution.

In addition to the surface area, particle size also affects the dissolution of nanoparticles. From the physicochemical properties outlined in Table 1, it can be observed that AgNPs have the smallest size diameter followed by AuNPs and TiO_2_ NPs have the largest particle diameter at 10 nm, 14 nm and 25 nm, respectively. The smaller the particle size the greater the availability of the surface area and this leads to increased chances of ion diffusion from the surface to the bulk fluid. It is for this reason that about 70% mass of Ag^+^ ions dissolved in simulated gastric fluid. Whereas the highest dissolution of Ti+ ions could only reach a maximum of 55% in simulated gastric fluid. These results are corroborated by those obtained by Hedberg et al. [50] and Murugadoss et al. [58].

Similar to particle size, particle surface functionalization is another factor that influences the dissolution kinetics of nanoparticles. It could be inferred that the addition of functional groups to the gold nanoparticles surface enhanced dissolution because the amine and carboxyl functional groups have better solubilizing properties compared to citrate stabilized AuNPs. Additionally, citrate was present on the AuNP surface as a stabilizing agent therefore can be easily displaced thereby encouraging nanoparticle agglomeration. These authors also concluded that the rate of dissolution depends on the type of functional group attached to the nanoparticle surface [59,60,61].

Of all the simulated fluids, synthetic seawater had the highest ionic strength and highest concentration of divalent cations such as Ca^2+^ and Mg^2+^ These divalent cations are known to induce particle aggregation by suppressing the electrostatic repulsive forces between the particle–particle interactions [30,56]. This leads to the reduction in the surface area to volume ratio thereby inhibiting dissolution from occurring. This would explain the low dissolution rates of nanoparticles exposed to synthetic seawater. In so far as particle functionalization is concerned, the presence of Polyethylene glycol (PEG) as a coating agent on the surface of functionalized gold nanoparticles (COOH-AuNPs) and (NH_2_-AuNPs) reduces particle agglomeration and this is attributed to the steric repulsive forces imparted by PEG on the nanoparticle surface. These results were corroborated by Botha et al. [44] and Breitner et al. [45].

Generally, when particles were exposed to simulated fluids, the dissolution was significantly lower in alkaline media than in acidic media. This is because acidic conditions, as evidenced by low pH values in gastric and phagolysosomal fluids, enable the oxidation of nanoparticles into ions, increasing their solubility and thus the likelihood of dissolution [24,62]. As a result, nanoparticles exposed to acidic media would be less stable and dissolve more readily than in alkaline media. This could explain why citrate stabilized gold nanoparticles, carboxyl functionalized gold nanoparticles and silver nanoparticles dissolved faster in highly acidic simulated gastric fluid and phagolysosomal fluid. Other researchers corroborate these results and have observed that particles exposed to simulated fluids characterized by alkaline conditions reach a point of zero charge and generally low amounts of ions get released under these circumstances [53,63].

## 5. Conclusions

This study investigated the biodurability and persistence of gold, silver and titanium dioxide nanoparticles using the continuous flow-through system. The dissolution kinetics of the nanoparticles were affected by pH, ionic strength, particle aggregation and agglomeration state, as well as surface functionalization. Results showed that all three types of nanoparticles had varying levels of biodurability/persistence; however, silver nanoparticles had the highest rate of dissolution in all simulated biological fluids and synthetic environmental media. This suggests that silver nanoparticles are more likely to have short-term health and environmental effects, which could be similar to those caused by dissolved Ag ions. Gold nanoparticles on the other hand may have the potential to cause both short-term and long-term health and environmental effects depending on their surface functionalization. PEGylated gold nanoparticles are more resistant to agglomeration than citrate-stabilized gold nanoparticles due to ligand-promoted processes that increase dissolution rates. Citrate-stabilized gold nanoparticles have low dissolution rates and can cause long-term health effects as they are more stable and persistent. Titanium dioxide nanoparticles have low dissolution rates, high stability and form agglomerates, making them particularly biodurable and biopersistent in aquatic environments and likely to cause long-term toxicity. To ensure the safety of workers, consumers and the environment, it is critical to study the biodurability and persistence of nanoparticles.

## Figures and Tables

**Table 1:** Chemical composition, pH and ionic strength of simulated fluids (Marques et al., 2011).

Chemical Composition (g 5 L^−1^)	BP	GF	GIF	IF	PSF	FW	SW
Bile salts	-	-	-	45 mL	-	-	-
Borax	-	-	-	-	-	-	0.17
Calcium chloride	1.46	-	-	2.49	-	-	-
Calcium chloride anhydrous	-	-	-	-	-	-	1.320
Calcium chloride dihydrate	-	1.84	-	-	0.14	-	-
Calcium sulphate anhydrous	-	-	-	-	-	0.37	-
Glycine	-	-	-	-	2.25	-	-
Magnesium chloride	-	1.015		0.95			47.5
Magnesium chloride hexahydrate	1.65		-	-	-	-	-
Magnesium sulphate anhydrous	-	-	-	-	-	0.037	-
Mucin	-	-	15 mg	-	-	-	-
Pancreatin	-	-	-	45 mL	-	-	-
Pepsin	-	-	5 mL	-	-	-	-
Potassium bromide	-	-	-	-	-	-	0.44
Potassium chloride	1.12	1.49	35	1.49		0.0025	3.05
Potassium hydrogen phthalate	-	-	1.215	-	20.43	-	-
Potassium phosphate dibasic trihydrate	1.15	-	-	-	-	-	-
Sodium acetate	-	4.76	-	-	-	-	-
Sodium chloride	40.17	30.09	14.61		33.25		105.1
Sodium hydrogen carbonate	1.77	13.02	-	-	-	0.06	0.85
Sodium hydrogen phosphate	-	0.71	-	-	0.171	-	-
Sodium sulphate	0.36				0.36		17.6
Sodium sulphate anhydrous	-	0.085	-	-	-	-	-
Strontium chloride	-	-	-	-	-	-	0.1
Tris(hydroxymethyl) aminomethane	30.59	-	-	-	-	-	-
Trisodium citrate dihydrate	-	0.485	-	-	-	-	-
Urea	-	-	-	1.5	-	-	-
1 M HCl	195 mL	-	-	-	-		-
Ionic strength (mol L^−1^)	0.15	0.17	0.16	0.16	0.34	0.05	3.5
pH	7.2	7.4	2.0	6.8	4.5	6.8	8.0

BP—Blood plasma; GF—Gamble’s fluid; GIF—Gastric fluid; IF—Intestinal fluid; PSF—Phagolysosomal fluid; FW—Freshwater; SW—Seawater.

**Table 2:** Physical–chemical description of AuNPs, AgNPs and TiO_2_ NPs.

Nanoparticles	Simulated Fluids	UV-Vis Absorption Wavelength		Surface Area	Particle Size Diameter	Crystallinity (XRD)
		[nm]		[m^2^/g]	[nm]	[%]
		Before	After			
Citrate-AuNPs	BP	520	549	25	14	None
Citrate-AuNPs	GF	520	549	21	14	None
Citrate-AuNPs	GIF	520	549	23	14	None
Citrate-AuNPs	IF	520	549	21	14	None
Citrate-AuNPs	PSF	520	549	20	14	None
Citrate-AuNPs	FW	520	549	22	14	None
Citrate-AuNPs	SW	520	549	20	14	None
COOH-AuNPs	BP	520	547	24	14	None
COOH-AuNPs	GF	520	547	23	14	None
COOH-AuNPs	GIF	520	547	24	14	None
COOH-AuNPs	IF	520	547	26	14	None
COOH-AuNPs	PSF	520	547	26	14	None
COOH-AuNPs	FW	520	547	24	14	None
COOH-AuNPs	SW	520	547	25	14	None
NH_2_-AuNPs	BP	520	540	22	14	None
NH_2_-AuNPs	GF	520	540	23	14	None
NH_2_-AuNPs	GIF	520	540	22	14	None
NH_2_-AuNPs	IF	520	504	22	14	None
NH_2_-AuNPs	PSF	520	504	20	14	None
NH_2_-AuNPs	FW	520	540	18	14	None
NH_2_-AuNPs	SW	520	540	20	14	None
AgNPs	BP	400	450	22	10	None
AgNPs	GF	400	450	22	10	None
AgNPs	GIF	400	400	18	10	None
AgNPs	IF	400	450	15	10	None
AgNPs	PSF	400	400	15	10	None
AgNPs	FW	400	450	26	10	None
AgNPs	SW	400	400	20	10	None
TiO_2_ NPs	BP	300	320	57	25	Mix rutile/anatase
TiO_2_ NPs	GF	300	320	58	25	Mix rutile/anatase
TiO_2_ NPs	GIF	300	320	56	25	Mix rutile/anatase
TiO_2_ NPs	IF	300	320	55	25	Mix rutile/anatase
TiO_2_ NPs	PSF	300	320	55	25	Mix rutile/anatase
TiO_2_ NPs	FW	300	320	59	25	Mix rutile/anatase
TiO_2_ NPs	SW	300	320	58	25	Mix rutile/anatase

**Table 3:** Comparison of the dissolution rates and half-times of AuNPs, AgNPs and TiO_2_ NPs in simulated fluids.

Nanoparticles	Simulated Fluids	Dissolution Rate k	Half-Time $t_{1/2}$	*p*-Value
		[ng/cm^2^/h]	[days]	
Citrate-AuNPs	BP	0.09	10	0.0621
Citrate-AuNPs	GF	0.08	8.6	0.1138
Citrate-AuNPs	GIF	0.08	8.6	0.2144
Citrate-AuNPs	IF	0.06	12.5	0.0720
Citrate-AuNPs	PSF	0.10	7.3	0.0591
Citrate-AuNPs	FW	0.06	11.5	0.0820
Citrate-AuNPs	SW	0.05	12.5	0.0931
COOH-AuNPs	BP	0.08	6.5	0.0656
COOH-AuNPs	GF	0.08	7	0.0809
COOH-AuNPs	GIF	0.10	5	0.0633
COOH-AuNPs	IF	0.06	9	0.0744
COOH-AuNPs	PSF	0.10	5.7	0.0537
COOH-AuNPs	FW	0.06	10	0.0644
COOH-AuNPs	SW	0.09	7.5	0.0937
NH_2_-AuNPs	BP	0.13	4	0.1151
NH_2_-AuNPs	GF	0.11	6	0.2413
NH_2_-AuNPs	GIF	0.06	10	0.0655
NH_2_-AuNPs	IF	0.09	7	0.0594
NH_2_-AuNPs	PSF	0.06	10	0.0742
NH_2_-AuNPs	FW	0.13	4	0.0894
NH_2_-AuNPs	SW	0.06	10	0.0942
AgNPs	BP	0.15	4	0.0021
AgNPs	GF	0.15	4	0.0008
AgNPs	GIF	0.18	2	0.0144
AgNPs	IF	0.10	7	0.0420
AgNPs	PSF	0.2	2	0.0231
AgNPs	FW	0.12	6	0.0320
AgNPs	SW	0.10	7	0.0231
TiO_2_NPs	BP	3.70 × 19^−05^	13.6	0.0778
TiO_2_NPs	GF	3.47 × 10^−05^	14.3	0.0898
TiO_2_NPs	GIF	3.63 × 10^−05^	14.1	0.0755
TiO_2_NPs	IF	3.67 × 10^−05^	14.2	0.0894
TiO_2_NPs	PSF	3.65 × 10^−05^	14.3	0.0842
TiO_2_NPs	FW	3.40 × 10^−05^	14.3	0.2329
TiO_2_NPs	SW	3.43 × 10^−05^	14.4	0.1142

## Data Availability

Data will be made freely available on request, it can be requested from mary.gulumian@NWU.ac.za.
